# Socio-demographic determinants of pregnancy termination among adolescent girls and young women in selected high fertility countries in sub-Saharan Africa

**DOI:** 10.1186/s12884-021-04064-1

**Published:** 2021-09-04

**Authors:** Bright Opoku Ahinkorah

**Affiliations:** School of Public Health, Faculty of Health, University of Technology Sydney, Sydney, Australia

**Keywords:** Pregnancy termination, Adolescent girls, Young women, Sub-Saharan Africa, Reproductive health, Global Health

## Abstract

**Background:**

Most of the unintended pregnancies that occur among adolescent girls and young women (AGYW) in sub-Saharan Africa (SSA) end up in pregnancy termination. In this study, the socio-demographic determinants of pregnancy termination among AGYW (aged 15–24) in selected countries with high fertility rates in SSA were examined.

**Methods:**

This was a cross-sectional analysis of data from the most recent Demographic and Health Surveys of nine countries in SSA. The countries included are Angola, Burkina Faso, Burundi, Chad, Gambia, Mali, Niger, Nigeria, and Uganda. A total of 62,747 AGYW constituted the sample size for the study. Fixed and random effects models were used to examine the determinants of pregnancy termination with statistical significance at *p* < 0.05.

**Results:**

Higher odds of pregnancy termination were found among AGYW aged 20–24, those who were cohabiting and married, those who listened to radio and watched television at least once a week and those who lived in communities with high literacy level. Conversely, the odds of pregnancy termination were lower among AGYW with three or more births and those with secondary/higher education.

**Conclusion:**

The socio-demographic determinants of pregnancy termination among AGYW in this study were age, level of education, marital status, exposure to radio and television, parity, and community literacy level. The findings provide the needed information for designing health interventions to reduce unwanted pregnancies and unsafe abortions in countries with high fertility rates in SSA. It is recommended that governments and non-governmental organisations in these countries should enhance sexuality education and regular sensitization of adolescent sexual and reproductive health programmes targeted at AGYW who are at risk of pregnancy termination.

## Background

One of the major reasons for the high fertility rates in most countries in sub-Saharan Africa (SSA) is that a considerable number of women in SSA are not contraceptive users [1–4]. Several obstacles to the use of contraception have been cited in a number of studies [2, 5, 6]. These obstacles include inadequate knowledge on family planning methods and where they can be accessed, low quality and limited availability of family planning services, and high cost of family planning methods, services, travel, and time. Other obstacles centre around fear of side effects, disapproval from partners and family members, and concerns about moral and social acceptability [2, 5, 6]. These obstacles are more prevalent in adolescent girls and young women (AGYW), and contribute significantly to the high unmet need for contraception among this cohort of women [2, 6]. This may explain the approximately 80 million mistimed and unplanned pregnancies, which occur in low-and middle-income countries, and constitute 40% of all pregnancies [7]. Majority of these mistimed and unplanned pregnancies end in abortions (40 million) and are responsible for the high burden of health and socio-economic challenges for many women and their families [7].

In SSA, previous studies on pregnancy termination among AGYW have identified socio-demographic factors such as age, ethnicity, parity, occupation, age at first sex, marital status, place of residence, and region as factors associated with pregnancy termination among AGYW [8–10]. These studies were done in countries with lower fertility rates compared to countries, which have higher fertility rates [11]. Despite evidence that women, particularly AGYW in countries with high fertility rates in SSA have high unmet need for contraception, leading to high unintended pregnancies and abortions [2, 6], studies that have examined the determinants of pregnancy termination among AGYW in countries with high fertility rates in SSA are scanty. Moreover, there has not been any pooled analysis of nationally representative data on the determinants of pregnancy termination among AGYW in countries with high fertility rates in SSA. This study, therefore, aims to fill this gap by examining the socio-demographic determinants of pregnancy termination among AGYW in selected countries with high fertility rates in SSA. This study is important because it provides the needed information for designing sexual and reproductive health interventions to reduce unwanted pregnancies and unsafe abortion in SSA.

## Methods

### Data source

**Fig. 1:**
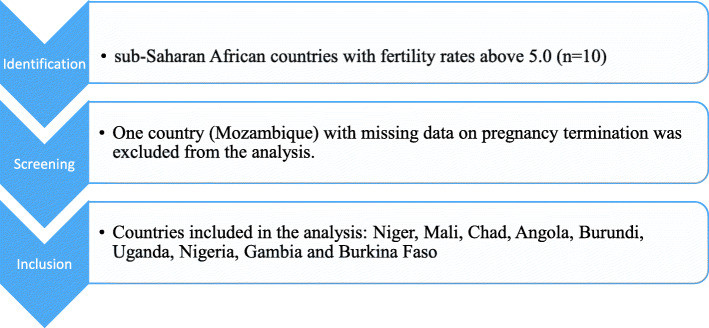
Inclusion and exclusion criteria

This was a cross-sectional analysis of data from the most recent Demographic and Health Surveys (DHSs) (2010–2019) of nine countries in SSA. The countries included are Angola, Burkina Faso, Burundi, Chad, Gambia, Mali, Niger, Nigeria, and Uganda (see Fig. 1). These countries were selected because they were ranked among the first ten countries in SSA with fertility rates above 5.0, a value that is higher than the rate of 4.7 in SSA and 2.4 globally [11]. The overarching objective of the DHSs is to generate demographic and health indicators that are nationally representative. They capture data on essential maternal and child health indicators, including pregnancy termination [12, 13].

### Sample and sampling strategy

A two-stage stratified sampling procedure was used in sampling the participants for the DHS. Detailed information on the two-stage sampling procedure exist in previous studies [12, 13]. A total of 62, 747 AGYW (aged 15–24) were included in this study. Table 1 has comprehensive information on the sample.

**Table 1:** Description of surveys and study sample

Country	Year of survey	Weighted sample (n)	Weighted sample (%)
Angola	2015–16	6498	10.36
Burkina Faso	2010	6598	10.51
Burundi	2016–17	7109	11.33
Chad	2014–15	6846	10.91
Gambia	2013	4496	7.17
Mali	2018	4003	6.38
Niger	2012	3806	6.07
Nigeria	2018	15,297	24.38
Uganda	2016	8093	12.90
Total	–	62,747	100

### Study variables

#### Outcome variable

The outcome variable in this study was pregnancy termination. Pregnancy termination in the DHS includes induced abortions, stillbirths and miscarriages. To derive this variable, survey participants were asked “have you ever had a pregnancy terminated?” Two responses emanated from this question “No” and “Yes”. These two responses were used to define the outcome variable in line with previous studies [14–16].

#### Explanatory variables

The explanatory variables were grouped into individual level factors (age, marital status, level of education, wealth quintile, frequency of reading newspaper/magazine, listening to radio and watching television, and parity) and contextual level factors (place of residence, community literacy level, and community socio-economic status). The selection of these variables was based on their significant associations with pregnancy termination in previous studies [14–16] and their availability in the DHS datasets used in this study. The various categories used to describe each of these variables are in Tables 2 and 3. This study also adopted the categorization of the variables from previous studies [6, 17].

**Table 2:** Distribution of pregnancy termination among adolescent girls and young women by the explanatory variables (Weighted)

Variables	Frequency (n)	Percentage (%)	Pregnancy termination		***p***-value
Individual level variables			Yes	No	
**Age**					*p* < 0.001
15–19	33,492	53.4	2.4	97.6	
20–24	29,255	46.6	8.5	91.5	
**Level of education**					*p* < 0.001
No Education	19,780	31.5	6.5	93.5	
Primary	16,604	26.5	6.2	93.8	
Secondary/Higher	26,363	42.0	3.7	96.3	
**Marital Status**					*p* < 0.001
Single	34,510	55.0	1.4	98.6	
Cohabiting	5870	9.4	12.1	87.9	
Married	22,367	35.7	9.4	90.6	
**Wealth quintile**					*p* < 0.001
Poorest	10,302	16.4	6.1	94.0	
Poorer	11,844	18.9	6.0	94.0	
Middle	12,164	19.4	5.7	94.3	
Richer	13,082	45.9	5.0	95.0	
Richest	15,355	24.5	4.1	96.0	
**Frequency of reading newspaper/magazine**					*p* < 0.001
Not at all	52,322	83.4	5.5	94.5	
Less than once a week	5242	8.4	3.9	96.1	
At least once a weak	5182	8.3	4.0	96.0	
**Frequency of listening to radio**					*p* < 0.001
Not at all	26,172	41.7	5.0	95.0	
Less than once a week	12,319	19.6	5.1	94.9	
At least once a week	24,256	38.7	5.6	94.4	
**Frequency of watching television**					*p* < 0.001
Not at all	35,436	56.5	5.8	94.2	
Less than once a week	8361	13.3	4.7	95.3	
At least once a weak	18,951	30.2	4.4	95.6	
**Parity**					*p* < 0.001
Zero births	35,551	56.7	2.3	97.7	
One birth	12,869	20.5	8.8	91.2	
Two births	8484	13.5	9.9	90.1	
Three or more births	5842	9.3	8.6	91.4	
Contextual level variables					
**Place of residence**					*p* < 0.001
Urban	23,133	36.9	4.3	95.7	
Rural	39,614	63.1	5.8	94.2	
**Community literacy level**					*p* < 0.001
Low	20,879	33.3	6.2	93.9	
Moderate	20,635	32.9	5.2	94.8	
High	21,233	33.8	4.4	95.6	
**Community socio-economic status**					*p* < 0.001
Low	31,750	50.6	5.9	94.1	
Moderate	8746	13.9	5.2	94.8	
High	22,251	35.5	4.3	95.7	

*P-values obtained from chi-square test of independence

**Table 3:** Multilevel logistic regression models for the socio-demographic determinants of pregnancy termination

Variables	Model 0 aOR[95%CI]	Model 1 aOR[95%CI]	Model 2 aOR[95%CI]	Model 3 aOR[95%CI]
**Fixed effects results**				
**Individual level factors**				
**Age**				
15–19		Reference		Reference
20–24		2.34^***^[2.12–2.59]		2.35^***^ [2.13–2.60]
**Level of education**				
No Education		Reference		Reference
Primary		1.39^***^[1.26–1.52]		1.13^*^[1.02–1.26]
Secondary/Higher		0.98 [0.88–1.10]		0.80^**^[0.71–0.91]
**Marital status**				
Single		Reference		Reference
Cohabiting		5.65^***^ [4.97–6.43]		6.29^***^ [5.60–7.20]
Married		6.22^***^ [5.39–7.18]		5.49^***^ [4.74–6.36]
**Wealth quintile**				
Poorest		Reference		Reference
Poorer		0.96 [0.86–1.08]		0.98 [0.87–1.10]
Middle		0.97 [0.86–1.09]		1.00 [0.88–1.13]
Richer		0.90 [0.79–1.02]		0.92 [0.80–1.05]
Richest		0.87 [0.75–1.00]		0.87 [0.72–1.04
**Frequency of reading newspaper/magazine**				
Not at all		Reference		Reference
Less than once a week		1.01 [0.86–1.19]		0.95 [0.81–1.12]
At least once a week		1.06 [0.89–1.27]		1.02 [0.85–1.23]
**Frequency of listening to radio**				
Not at all		Reference		Reference
Less than once a week		1.16^**^ [1.03–1.29]		1.18^**^ [1.05–1.32]
At least once a week		1.22^***^ [1.18–1.34]		1.20^***^ [1.09–1.32]
**Frequency of watching television**				
Not at all		Reference		Reference
Less than once a week		0.96 [0.85–1.09]		1.00 [0.87–1.14]
At least once a week		1.08 [0.97–1.20]		1.13^*^ [1.01–1.28]
**Parity**				
Zero births		Reference		Reference
One birth		1.13^*^ [1.01–1.27]		1.10 [0.98–1.23]
Two births		0.94 [0.82–1.06]		0.89 [0.78–1.01]
Three or more births		0.73^***^[0.64–0.85]		0.68^***^[0.59–0.80]
**Contextual level factors**				
**Place of residence**				
Urban			Reference	Reference
Rural			1.20^**^[1.08–1.33]	1.07 [0.95–1.20]
**Community literacy level**				
Low			Reference	Reference
Moderate			0.91^*^ [0.83–0.99]	1.10 [1.00–1.21]
High			0.80^***^[0.72–0.90]	1.18^*^ [1.04–1.34]
**Community socio-economic status**				
Low			Reference	Reference
Moderate			0.89 [0.80–1.00]	0.98 [0.86–1.10]
High			0.93 [0.82–1.04]	1.09 [0.94–1.26]
**Random effects results**				
PSU Variance(95% CI)	0.06 (0.03–0.10)	0.04 (0.02–0.09)	0.05 (0.03–0.10)	0.03 (0.01–0.08)
ICC	0.2	0.01	0.02	0.01
LR Test	χ^2^ = 15.4, *p* < 0.001	χ^2^ = 6.2, *p* < 0.05	χ^2^ = 14.2, *p* < 0.001	χ^2^ = 4.0, *p* < 0.05
Wald χ^2^	Reference	1982.9^***^	87.6^***^	2126.3^***^
Model fitness				
Log-likelihood	−12,320.6	−10,945.1	−12,275.7	−10,855
AIC	24,645.3	21,930.2	24,565.3	21,776.4
Sample size	62,747	62,747	62,747	62,747
Number of clusters	1391	1391	1391	1391

Exponentiated coefficients; 95% confidence intervals in brackets; ^*^p < 0.05, ^**^p < 0.01, ^***^p < 0.001

*PSU* Primary Sampling Unit, *ICC* Intracluster correlation coefficient, *LR Test* Likelihood ratio Test, *AIC* Akaike’s Information Criterion, *aOR* adjusted Odds Ratios, *CI* Confidence Interval

Model 0 is the null model, a baseline model without any explanatory variable

Model 1 is adjusted for individual level variables

Model 2 is adjusted for contextual level variables

Model 3 is the final model adjusted for all explanatory variables and survey country

### Data analyses

Using Stata 14.0, the analysis was performed by first calculating the prevalence of pregnancy termination and describing the characteristics of the participants using frequencies and percentages. Next, the distribution of the explanatory variables across pregnancy termination was done using Chi-square test with a statistical significance at *p* < 0.05. Finally, a mixed effects analysis (fixed and random effects) was performed to examine determinants of pregnancy termination using four models (Model 0, 1, 2, and 3). Log likelihood and Akaike’s Information Criterion (AIC) tests were used to check for model fitness while variations between models were assessed using the Intracluster Correlation Coefficient (ICC). Odds ratio with 95% confidence intervals (CIs) were used to present the fixed effects results. Variance inflation factor (VIF) was used to check for multi-collinearity and there was no evidence of multicollinearity. Sample weights were applied to all distributions and correction for the complex survey design was considered.

## Results

### Descriptive results

**Fig. 2:**
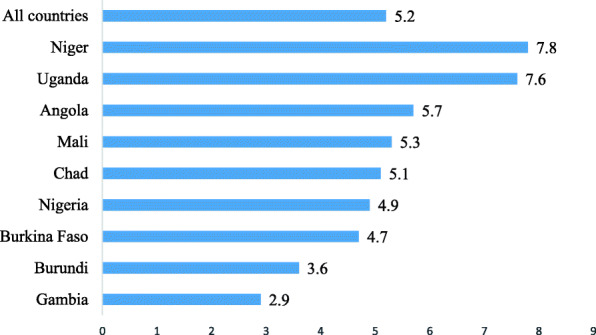
Proportion of adolescent girls and young women who had experienced pregnancy termination in the selected countries with high fertility in sub-Saharan Africa

Approximately 5% of the AGYW reported pregnancy termination, with the higher prevalence in Niger (7.8%) and a lowest prevalence in Gambia (2.9%) (Fig. 2).

The distribution of pregnancy termination among AGYW in the selected high fertility countries in SSA by the explanatory variables is presented in Table 2. At the individual level, the modal categories for pregnancy termination were found among respondents aged 20–24 (8.5%), those with no formal education (6.5%), and those who were cohabiting (12.1%). Similarly, pregnancy termination was frequent among AGYW with poor wealth quintile (6.0%), those who never read newspaper/magazine (5.5%), those who listened to radio at least once a week (5.6%), those who never watched television (5.8%), and those with two births (9.9%). With the community level factors, the modal categories for pregnancy termination were found among AGYW who lived in the rural areas (5.8%), those who lived in communities with low literacy level (6.2%), and those who lived in communities with low socio-economic status (5.9%).

### Socio-demographic determinants of pregnancy termination

In terms of the random effects results, a log likelihood of − 10,855 and an AIC of 21,776.4, was an indication that the best fit model was Model 3. With the fixed effects results for the individual level factors, higher odds of pregnancy termination were found among AGYW aged 20–24, AGYW who were cohabiting and married, those who listened to radio and watched television at least once a week. Conversely, the odd of pregnancy termination was lower among AGYW with three or more births and those with secondary/higher education. In terms of the community level factors, AGYW in communities with high literacy had higher odds for pregnancy termination than those in communities with low literacy level.

## Discussion

The determinants of pregnancy termination among AGYW in selected countries with high fertility in SSA were examined in the current study. Respondents aged 15–19 and those who were single had higher odds of pregnancy termination. A previous study in SSA observed that pregnancy termination is low among adolescent girls and never married women [15]. Barriers to accessing sexual and reproductive health services, including contraception could be the reasons for this finding [18–20]. Specifically, AGYW in most countries in SSA are often denied access to family planning services due to negative socio-cultural norms and this increases their tendency of experiencing unintended pregnancies, which could end in abortion [21, 22]. Apart from abortion, miscarriages and stillbirths, which also constitute other forms of pregnancy termination have been found to be higher among never married adolescent girls due to stigma, which affect their emotional and psychological health and low utilization of antenatal care services [23–25].

Pregnancy termination was high among AGYW with no births compared to those with four or more births. Similar findings have been obtained in Ghana and Mozambique [14]. Possibly, AGYW with no pregnancy history might not have the readiness to give birth, especially when they are young and not married and hence may opt to have their pregnancies terminated while others may lose their pregnancies through miscarriages and stillbirths [16].

AGYW with secondary/higher education also had lower odds of terminating their pregnancies compared to those with no formal education. In terms of the association between education and pregnancy termination, similar findings were obtained by Dickson, Adde [14] and Seidu, Ahinkorah [16]. Since pregnancy termination in the current study includes stillbirths and miscarriages, it is possible that AGYW with secondary/higher education may have the knowledge required to prevent these adverse pregnancy outcomes from occurring [26]. Moreover, higher levels of education may expose AGYW to the risks associated with induced abortions and hence may reduce their involvement in them. On the contrary, studies in China [27] and Ghana [28] found pregnancy termination to be high among women with higher educational level and argued that educated women may have pregnancies that interfere with their education and hence may decide to terminate those pregnancies. These findings may confirm the findings of the current study where AGYW who lived in high literacy communities had increased odds of pregnancy termination. In high literate communities, AGYW may have access to information on pregnancy termination and hence may act on the knowledge gained to seek abortion services. Such information may be available on radio and television, which have been found in the current study to increase the likelihood of pregnancy termination among those who are exposed compared to those who are not exposed. The disparities in findings could be due to the type of data used and the study population. While the current study and the studies by Dickson, Adde [14] and Seidu, Ahinkorah [16] used nationally-representative secondary data from the DHS, the studies that contradict the findings of the current study used primary data and focused on sub-sections of the population.

### Strengths and limitations

This study is supported by the use of nationally representative large sample and reliable data. This makes it possible to generalise the findings to AGYW in other countries with high fertility. Notwithstanding, due to the cross-sectional nature of the surveys, this study cannot draw causal interpretations between the factors and pregnancy termination, at best only associations can be drawn. Secondly, pregnancy termination was self-reported and could have been prone to recall bias. Also, due the socio-cultural norms around pregnancy termination among AGYW, the respondents in this study may have under-reported pregnancy termination due to fear of stigma. Moreover, the data used were from different years and this may affect the generalizability of the findings. Finally, the sampling time is different between countries, which may cause a bias in comparing the findings between countries.

## Conclusion

The socio-demographic factors associated with pregnancy termination among AGYW in this study were age, level of education, marital status, exposure to radio and television, parity, and community literacy level. The findings provide the needed information for designing health interventions to reduce unwanted pregnancies and unsafe abortions. It is recommended that governments and non-governmental organisations in these countries should enhance sexuality education and regular sensitization of adolescent sexual and reproductive health programmes targeted at AGYW who are at risk of pregnancy termination. Such interventions will contribute to the achievement of the Sustainable Development Goal 3.1 that seeks to reduce the global maternal mortality ratio to fewer than 70 per 100,000 live births by 2030.

## Data Availability

The data for this study can be accessed on https://dhsprogram.com/data/available-datasets.cfm.

## Abbreviations

AIC: Akaike’s Information Criterion
aOR: Adjusted odds ratio
AGYW: Adolescent girls and young women
DHS: Demographic and Health Survey
ICC: Intracluster Correlation Coefficient
SSA: Sub-Saharan Africa
VIF: Variance Inflation Factor
